# Medical History from the Earliest Times

**Published:** 1892-05-21

**Authors:** E. T. Withington


### Medical History from the Earliest Times.

By E. T. Withington, M.A., M.B. Oxon.

### IX.—The Philosophers—Democedes—The Æsclepiadæ.

Celsus tells us in a well-known passage that Hippocrates first separated medicine, not from priestcraft, but from philosophy. Though several of the older philosophers are said to have rendered aid in epidemics, and to have treated their friends and pupils medically, of more importance are the influences which their views of nature had upon medical theories, and their early attempts at anatomy. These were chiefly concerned, as might be expected, with departments in which mind and body seem to be in closest contact, the organs of special sense, and the processes of generation, sleep, and death. Pythagoras (B.C. 580-510) tended his followers when sick, and went to Delos to cure his master, Pherecydes, of phthiriasis. He also established a strict system of dietetics, and thought highly of the curative powers of music, especially in diseases of spring-time, and when the mind was affected. His ideas of the mystical value of numbers may have had something to do with the Hippocratic doctrine of critical days, and his followers, we are told, paid much attention to medicine, preferring always the most soothing forms of treatment, and avoiding, as far as possible, the knife and cautery.

Pythagoras spent the latter part of his life at Croton, a great Achæan colony in South Italy, famous in antiquity for healthy situation, fair women, and strong men. According to Herodotus, the most famous physicians of his day came from this city, and it has been suggested, though without much evidence, that they were Pythagoreans. Of the two whose names have come down to us, Alcmæon and Democedes, the former was rather a philosopher than a physician, though his doctrines differ widely from those of Pythagoras. Alcmæon is said to have been the first Greek anatomist, and to have dissected the eyes and ears of animals, discovering the optic nerve and the Eustachian canal. He explained hearing by the hollow bone behind the ear—"for all hollow things are sonorous." Taste by the warmth, moisture, and softness of the tongue, while odours are perceived directly by the soul, which resides in the brain. Sleep and waking he held to be due to the ebb and flow of the blood in the great vessels, the cessation of which causes death. Specially interesting is his description of disease as a disturbance of the equilibrium of the vital activities by the predominance of some one of them, a definition peculiarly consonant with the views of the Greeks, who loved to see harmony and proportion in all things, and to whom a "monarchia," or dominance of one, was the root of all evil both to the state and the individual.

Democedes (about B.C. 520) is the first physician of whom we have a trustworthy history. Herodotus relates that, having migrated from Croton to Ægina, Democedes so excelled his colleagues that he was chosen public medical officer at a salary of one talent (£240) per annum. In the following year he went in the same capacity to Athens, where he received £406, and finally was invited to Samos by Polycrates, who paid him £480 a year. In estimating these sums we must remember that money had then many times its present purchasing power, and that the salary of an Athenian ambassador, in the days of Aristophanes, was two drachmæ (eighteen pence) a day, less than one-twelfth the amount given to Democedes. On the treacherous murder of Polycrates by the Persians, Democedes became a slave, and was finally brought to the Court of Darius, where he cured the King of a sprained ankle, and rescued the Egyptian surgeons, who had failed to do so, from impalement. Soon afterwards he successfully treated the Queen for a mammary abscess, and received from the grateful monarch all he could ask for except liberty. But to an ancient Greek liberty was the half of existence, and to obtain it Democedes offered to act as a guide to Persian spies, and, perhaps, to win over the leading men of his country to the Persian cause. On reaching Croton he, of course, refused to accompany his escort any further, and with the wealth given him by Darius, he obtained a beautiful wife, the daughter of Milo the athlete. Later writers tell us that his fellow-townsmen, so far from being indignant at conduct calculated to bring upon them the wrath of the great King, elected Democedes their chief magistrate, and it may have been at his instigation that Phäyllus of Croton fitted out the trireme which singly and honourably represented the colonies of Magna Græcia on the great day of Salamis.

The story of Democedes is important as showing that there was a well-established and extremely well paid public medical service in Greek cities in the sixth century (B.C), and there are indications of this at least a century earlier. Thus Diodorus tells us that Charondas, the lawgiver (B.C. 620), was greater than those who came before him, inasmuch as the mind exceeds the body, for he ordained free education in the cities for which he legislated, while they only provided a free medical service. The still earlier laws of Zaleucus forbade anyone to drink undiluted wine, except by order of a physician.

Democedes may have gained some of his skill in the gymnasia of Croton, and it is certain that the gymnasiarchs or trainers, often invaded the domain of medicine, especially in the departments of dietetics, the use of ointments, and the treatment of sprains and dislocations. Some, like Herodicus of Selymbria, went still further, and declared that all diseases, even acute fevers, might be treated by appropriate exercises.

But we must pass to another medical philosopher, hardly less famous than Pythagoras, Empedocles of Agrigentum (B.C. 490-430). The following is from a fragment of his poem, "On Nature":—

> Listen, first, while I sing the four-fold root of creation,
> Fire, and water, and earth, and the boundless height of the æther,
> For therefrom is begotten what is, what was, and what shall be.

Substituting air for æther this is the doctrine of the four elements, which Empedocles introduced into philosophy, and which, with the corresponding four qualities, heat, cold, moisture, and dryness, and the four humours, blood, phlegm, black and yellow bile, lies at the base of Greek medical theories. Empedocles discovered the labyrinth of the ear, and explained sound by the impact of the air upon it, as upon a drum.

He is also said to have cured a woman who lay in a trance thirty days, and to have removed the unhealthiness of certain localities by blocking up a mountain cleft and diverting the course of a river.

Empedocles dedicates his poem, "On Nature," to Pausanias of Gela, "the Æsclepiad," which, if not a mere poetical expression, is the first mention of a most important class of physicians, to which Hippocrates himself belonged—the Æsclepiadæ. Theopompus, the annalist (B.C. 350), tells us that the physicians of Cos and Cnidus were descended from Æsculapius through Podalirius, that they came from Syrnum in Caria, and that they were called Æsclepiadæ. The idea that these Æsclepiadæ were priests, supported by no ancient authority, is now generally abandoned, and with it the theory that Greek medicine originated in the temples of Æsculapius.

The Greeks, in fact, had no sacerdotal class, the depository of divine mysteries, as in Egypt and India; their priests were merely guardians of shrines who were usually appointed by the state. Physicians, as we have seen, were quite distinct from priests in the days of Homer, and we have been able to trace the existence of lay practitioners in Greek cities to a period probably nearly as ancient as the deification of Æsculapius.

But if the Æsclepiadæ were not priests what were they? We know little more than what is mentioned above; but in all probability they formed a guild or corporation, the leaders of which traced their descent to Æsculapius, but which admitted others to its ranks upon their taking an oath of membership. The text of this oath has come down to us among the writings attributed to Hippocrates, and though space will not permit even an abstract of its contents, in dignity of tone and high morality it is well worthy of its supposed author.

To conclude. We find in ancient Greece besides physicians proper, three classes of men connected with the healing art, priests, philosophers, and gymnastic trainers, corresponding roughly to our faith-healers, pure physiologists, and bone-setters respectively. The profession of medicine was separate from, though to some extent indebted to, all three, but it was most separate from, and least indebted to, the priests.

